# Herbivory Differentially Affects Plant Fitness in Three Populations of the Perennial Herb *Lythrum salicaria* along a Latitudinal Gradient

**DOI:** 10.1371/journal.pone.0135939

**Published:** 2015-09-01

**Authors:** Lina Lehndal, Jon Ågren

**Affiliations:** Plant Ecology and Evolution, Department of Ecology and Genetics, Evolutionary Biology Centre, Uppsala University, Uppsala, Sweden; Helmholtz Centre for Environmental Research (UFZ), GERMANY

## Abstract

Herbivory can negatively and selectively affect plant fitness by reducing growth, survival and reproductive output, thereby influencing plant population dynamics and evolution. Latitudinal variation in intensity of herbivory is common, but the extent to which it translates into corresponding variation in effects on plant performance is still poorly known. We tested the hypothesis that variation in the fitness-consequences of herbivory mirror differences in intensity of herbivory among three natural populations of the perennial herb *Lythrum salicaria* along a latitudinal gradient from southern to northernmost Sweden. We documented intensity of herbivory and examined its effect on survival, growth and reproductive output over two years by experimentally removing herbivores with insecticide. The intensity of herbivory and the effects of herbivory on plant fitness were strongest in the southern population, intermediate in the central population and weakest in the northern population. The mean proportion of the leaf area removed ranged from 11% in the southern to 3% in the northern population. Herbivore removal increased plant height 1.5-fold in the southern and 1.2-fold in the central population, the proportion plants flowering 4-fold in the southern and 2-fold in the central population, and seed production per flower 1.6-fold in the southern and 1.2-fold in the central population, but did not affect plant fitness in the northern population. Herbivore removal thus affected the relative fecundity of plants in the three populations: In the control, seed output per plant was 8.6 times higher in the northern population compared to the southern population, whereas after herbivore removal it was 2.5 times higher in the southern population. The results demonstrate that native herbivores may strongly affect the demographic structure of *L*. *salicaria* populations and thereby shape geographic patterns of seed production. They further suggest that the strength of herbivore-mediated selection varies among populations and decreases towards the north.

## Introduction

Herbivory can negatively and selectively affect plant performance by reducing growth, survival and reproductive output, thereby influencing both plant population dynamics and evolution [1,2]. Yet, the causes and consequences of variation in herbivory among populations remain poorly known. In many plant species, a decrease in the intensity of herbivory from south to north has been documented along latitudinal gradients in the northern hemisphere [3–7]. This has been linked to a general decrease in herbivore species diversity and abundance away from the equator [7]. Abiotic factors may limit the distribution of insect herbivores more than that of their host plants [8,9], for instance if the plant is more tolerant to harsh winter conditions and a short growing season than are the herbivores. Moreover, the shorter growing season at northern latitudes may be associated with reduced plant growth, which could contribute to spatial variation in intensity of herbivory if larger plants are more attractive to herbivores as has been observed in some species [10–12].

If herbivory decreases with latitude, this suggests that also the influence of herbivory on plant fitness and selection on resistance traits should decrease with latitude. Consistent with this idea, allocation to defence decrease with latitude in some systems [13–15]. However, the fitness-consequences of herbivory depend not only on intensity of herbivory but also on plant tolerance to herbivory, which may vary with environmental conditions [16–19]. An experimental approach is therefore required to determine whether the effects of herbivory on plant fitness vary along latitudinal gradients, but this has so far not been attempted.

We examined geographic variation in intensity of herbivory and its effect on plant fitness in the perennial herb *Lythrum salicaria*. In a previous study, intensity of damage tended to decrease with latitude among 12 natural populations of *L*. *salicaria* located along a latitudinal gradient in Sweden, and so did plant resistance to herbivory in a common garden-experiment including the same populations [14]. Here, we tested the hypothesis that differences in the fitness-consequences of herbivory mirror differences in intensity of herbivory along the latitudinal gradient, and thus decrease from south to north. In three populations sampled along the latitudinal gradient, we documented natural levels of herbivory, and we experimentally quantified its effects on three components of plant fitness (survival, size and reproductive output) by excluding herbivores with insecticide. Because in perennial plants, herbivory may reduce plant fitness also in the year following damage [20,21], we quantified effects also on size and reproductive output in the year following the experimental treatment.

Specifically, we asked: (1) Is plant fitness reduced by herbivory and, if so, which fitness components are affected? (2) Does among-population variation in effects of herbivory on plant fitness reflect differences in intensity of herbivory along a latitudinal gradient? (3) Does damage from insect herbivores affect plant performance also in the year following damage?

## Materials and Methods

### Study species

Purple loosestrife, *Lythrum salicaria* (L., Lythraceae), is a tristylous perennial herb, native to Eurasia and invasive in North America and Australia [22]. It is found in a variety of wetland habitats throughout its range. One or several above-ground shoots are produced by each plant, developing from winter buds formed on the rootstock in the previous year. Flower buds are produced in leaf nodes in the upper part of the flowering shoots. In Sweden, *L*. *salicaria* flowers for six to eight weeks in July and August, and the seeds mature six to eight weeks after flowering [23].

In Sweden, two specialist leaf beetles, *Galerucella calmariensis* L. and *Galerucella pusilla* L. (Coloptera: Chrysomelidae) are the main herbivores on *L*. *salicaria*. The monophagous leaf beetles overwinter in the soil as adults and emerge in the spring when aboveground shoots are developing. After emergence, the adult beetles mate and lay eggs on leaves and stems. After seven to ten days, the larvae hatch and feed for two to three weeks before pupating in the soil. Adult *G*. *calmariensis* and *G*. *pusilla* mainly feed on leaves, and larvae feed on leaves and flower buds [24]. Herbivory of *G*. *calmariensis* and *G*. *pusilla* on purple loosestrife can cause extensive damage to the host plant, and the beetles are currently used as biological control agents in North America [25].

The two species have similar life histories, but adults of *G*. *calmariensis* are slightly larger than *G*. *pusilla* adults [26]. The two species coexist in southern Sweden, including the southern site of the present study (Forsmark), but in northern Sweden only *G*. *calmariensis* is present (central and northern sites of the present study, Vitskärsudden and Skagsudden; [26]). Furthermore, the northernmost part of Sweden was free from *Galerucella* herbivory until recently; at Skagsudden, beetles were observed for the first time in 2011 [14].

The weevil *Nanophyes marmoratus* Goeze (Coleoptera: Curculionidae) is the main seed predator on *L*. *salicaria* in Sweden, and occurs across the study area. It feeds solely on *L*. *salicaria* and has a univoltine life cycle. The adults emerge in early summer on young *L*. *salicaria* shoots, and feed on young leaves and later also on flower buds. The females oviposit on young flower buds, and the emerging larva consumes the reproductive parts of the flower and pupates at the bottom of the bud [27].

### Study sites

**Fig 1:**
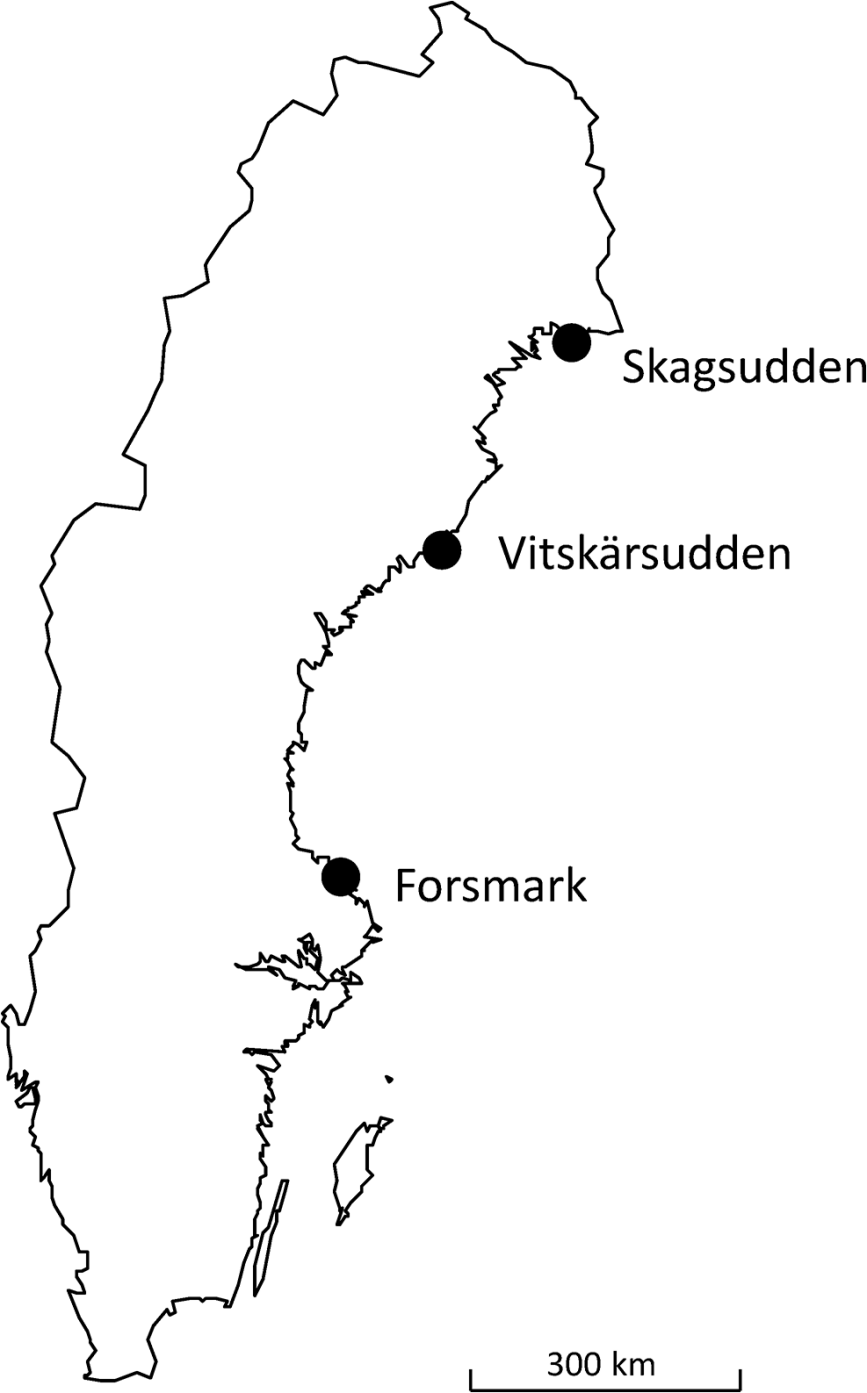
Map of Sweden showing the locations of the three *Lythrum salicaria* study populations.

The three study populations (Forsmark 60.40°N, 18.22°E, Vitskärsudden 63.65°’N, 20.29°E and Skagsudden 65.70°N, 23.10°E) were located along a latitudinal gradient from the mid to the northernmost part of the Swedish east coast, separated by approximately 250 to 650 km (Fig 1). Along the gradient, both yearly mean temperature and the length of the growing season decrease from south to north (Forsmark: 5–6°C, 180–190 days; Vitskärsudden: 2–3°C, 150–160 days; Skagsudden: 1–2°C, 140–150 days) [28]. At each site, we marked 200 plants per population in May 2013.

The field studies did not involve endangered or protected species, and field work was conducted on public land, on which no special permit is required for this kind of study.

### Herbivore removal experiment

To determine the effects of herbivory on plant survival, growth and reproductive output, we performed an herbivore-removal experiment. In each population, half of the plants were randomly assigned to an herbivore-removal treatment, in which plants were sprayed with an insecticide (Calypso, Bayer Garden, with the active substance Thiacloprid) on five occasions during June and July 2013, following user recommendations. The remaining plants served as control and were sprayed with an equal amount of water. In a previous greenhouse experiment, the insecticide Calypso did not affect growth and flowering of *L*. *salicaria* in the absence of herbivores [14].

### Data collection

Before the start of the experiment, we recorded plant height in spring 2013 in the Forsmark (May 17), Vitskärsudden (May 29), and Skagsudden (May 31) populations (hereafter referred to as early-season plant height). At the time of fruit maturation, on August 28 (Skagsudden), August 29 (Vitskärsudden) and September 3 (Forsmark), we recorded for each plant survival, height (hereafter referred to as late-season plant height), reproductive status (flowering or not flowering), the number of flower-producing shoots produced by reproductive plants, and the proportion of the total leaf area consumed by herbivores (leaf damage).

One haphazardly chosen floral shoot from each reproductive plant was collected and brought to the laboratory. In the laboratory, we recorded the number of leaf nodes producing flowers and total fruit production of collected shoots. To estimate flower production, we scored the number of flowers formed in each of five haphazardly chosen flower-producing leaf nodes. Number of flowers was obtained by adding the number of scars left by aborted flowers and fruits to the number of mature fruits. For each flowering plant, total flower production was estimated as number of flowering shoots × number of flower-producing leaf nodes on the collected shoot × mean number of flowers per leaf node, and total fruit production as number of flowering shoots × number of fruits on the collected shoot. We counted the number of seeds in five intact (non-dehisced) fruits per plant. For each plant, total seed production was estimated as number of fruits × mean number of seeds per fruit, and the number of seeds per flower was estimated as total seed production/number of flowers. For each population and treatment, mean female reproductive success was estimated as the mean number of seeds per plant, where plants failing to produce flowers were given the value 0.

During the winter of 2013/2014, storms removed the markings from more than half of the plants at Vitskärsudden; in the Forsmark and Skagsudden populations markedly lower proportions of plants lost their markings (6% and 14%, respectively). Because of the strong reduction in sample size, we excluded the Vitskärsudden population from the examination of treatment effects on performance in the second year.

In 2014, we recorded survival and plant height on May 28 (Forsmark), and June 11 (Skagsudden). At fruit maturation, we scored survival, height and fruit production as in the previous year. For logistic reasons, we did not score seed production. However, the number of fruits and the number of seeds per reproductive plant are strongly correlated in *L*. *salicaria* (Spearman rank correlation, *r*_*s*_ = 0.93, *n* = 359, *P* < 0.0001, data from 2013), and we used number of fruits produced per plant (including plants that did not flower) as an estimate of female reproductive success in the year following the experimental treatment.

### Statistical analysis

All statistical analyses were conducted using R statistical software version 3.1.1 [29].

We used one-way ANOVA to determine whether natural levels of herbivory varied among populations.

The effects of population and herbivore removal on the proportion of leaf area removed, early- and late-season plant height and most measures of reproductive performance were analysed with two-way ANOVA. In ANOVA models, the proportion of leaf area removed was arcsine square-root transformed, plant height (both early- and late-season) was square-root transformed and number of flowers per reproductive plant and measures of female reproductive success were log-transformed prior to analyses to meet assumptions of homoscedasticity and normally distributed residuals. Effects on flowering status (flowering vs. not flowering) were analysed with generalized linear models with binomial error distributions.

When statistically significant interactions between population and herbivore removal were detected, we specified a priori contrasts using the “contrast” package in R, to determine in which populations a significant treatment effect was detected.

## Results

### Variation in damage of control plants

**Fig 2:**
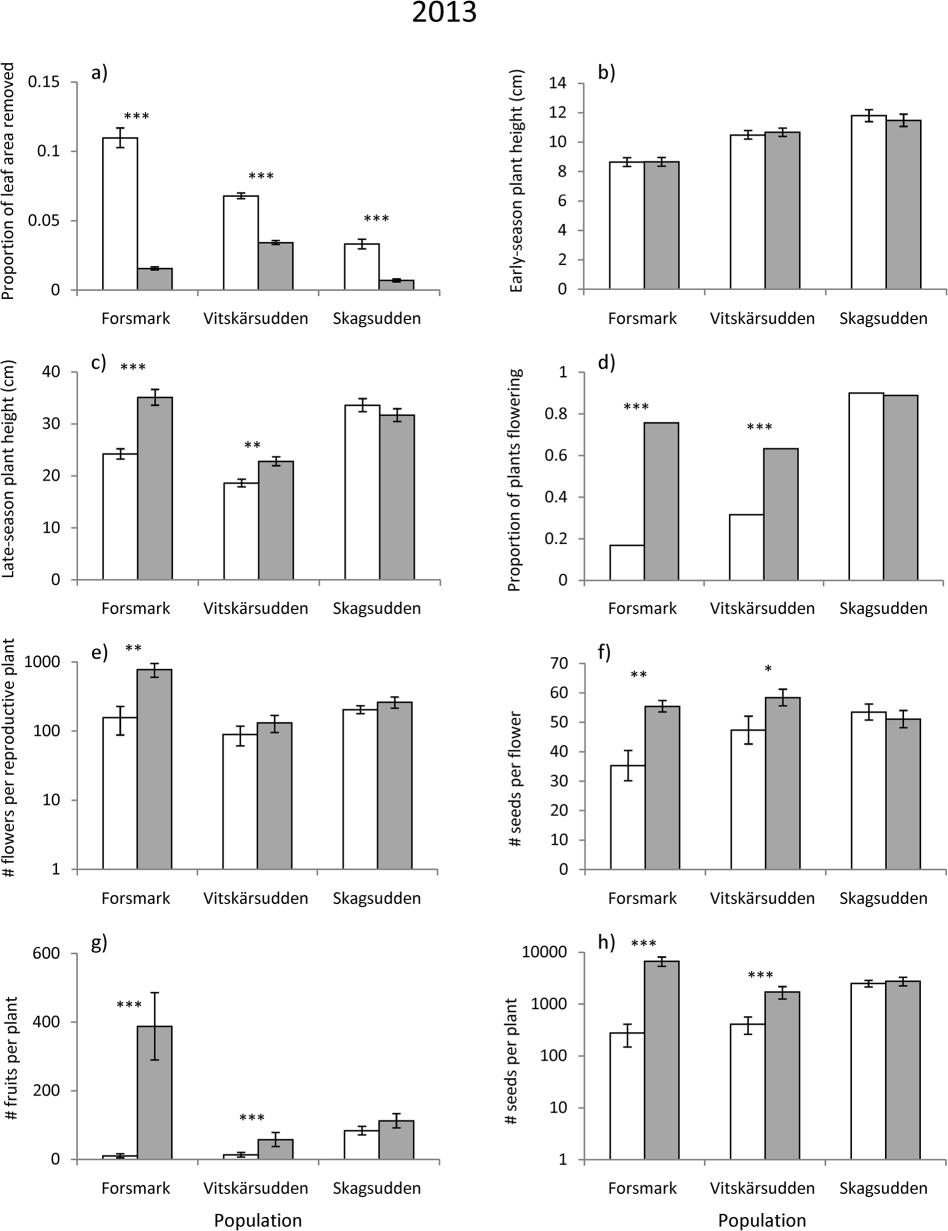
Herbivory, size and measures of reproductive performance of control plants (open bars) and plants treated with insecticide (filled bars) in three *Lythrum salicaria* populations (the southern Forsmark, the central Vitskärsudden, and the northern Skagsudden) in Sweden in the year of the experimental treatment. Population means ± SE are given for continuous variables and proportions for flowering status: (a) proportion of leaf area removed, (b) early-season plant height, (c) late-season plant height, (d) proportion of plants flowering, (e) number of flowers per reproductive plant, (f) number of seeds per flower, (g) number of fruits produced per plant including vegetative plants and (h) number of seeds produced per plant including vegetative plants. Significant differences between control plants and plants from which herbivores where removed are indicated. * *P* < 0.05, ** *P* < 0.01, *** *P* < 0.001.

**Fig 3:**
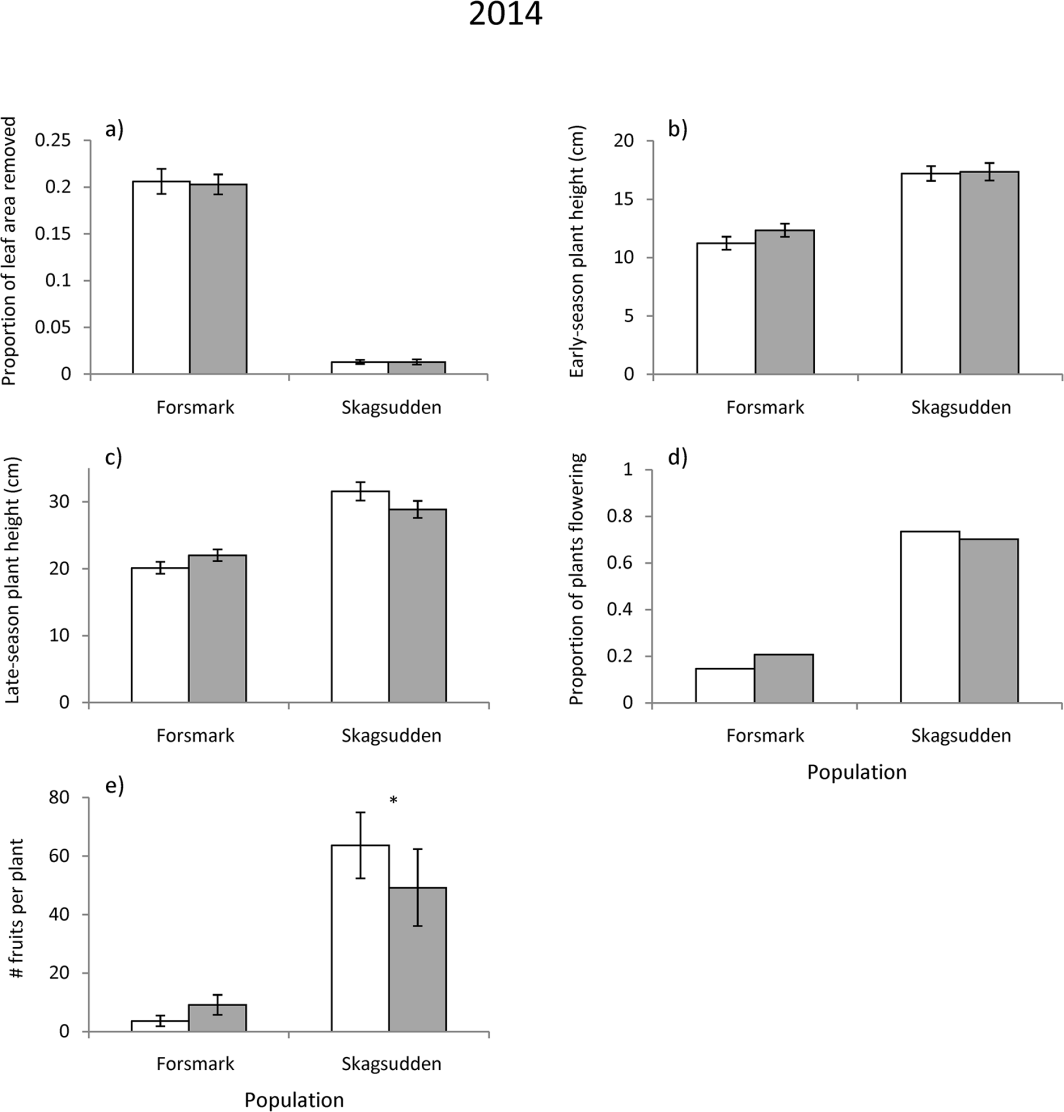
Herbivory, size and measures of reproductive performance of control plants (open bars) and plants treated with insecticide in the previous year (filled bars) in two *L*. *salicaria* populations (the southern Forsmark and the northern Skagsudden) in Sweden. Population means ± SE are given for continuous variables and proportions for flowering status: (a) proportion of leaf area removed, (b) early-season plant height, (c) late-season plant height, (d) proportion of plants flowering and (e) number of fruits produced per plant including vegetative plants.

The intensity of herbivory varied significantly among populations in the control treatment. In the year of the experimental treatment, the proportion of leaf area removed by herbivores was highest in the southern population, Forsmark (11%), intermediate in the central population, Vitskärsudden (7%) and lowest in the northern population, Skagsudden (3%; *F*_2,288_ = 72.40, *P* < 0.0001, Tukey: southern > central > northern population, Fig 2A). In the following year, the intensity of herbivory was even greater in the southern population (21%), but again very low in the northern population Skagsudden (1%; *F*_2,168_ = 550.05, *P* < 0.0001, Tukey: southern > northern population, Fig 3A).

### Effects of herbivore removal

The insecticide treatment reduced the proportion of leaf area removed by herbivores in all three populations in the year of the treatment, but had no effect on damage in the following year (Tables 1 and 2, Figs 2A and 3A).

**Table 1:** Effects of population and herbivore removal on damage from herbivores and fitness components in three *Lythrum salicaria* populations in the year of the experimental treatment (2013). Flowering status (flowering vs. not flowering) was analysed with a generalized linear model (*χ2* given); the other response variables were analysed with ANOVA (*F*-ratios given). The effect of herbivore removal was analysed separately by population with contrasts when a significant population × treatment interaction was detected. Significant effects are in bold.

					Forsmark		Vitskärsudden		Skagsudden	
Response variable	Source of variation	df	*F / χ2*	*P*	*t*	*P*	*t*	*P*	*t*	*P*
Proportion of leaf area removed	Population	2,580	101.75	**<0.0001**						
	Herbivore removal	1,580	327.62	**<0.0001**	18.1	**<0.0001**	7.18	**<0.0001**	9.49	**<0.0001**
	Pop × Herb rem	2,580	33.78	**<0.0001**						
Early-season plant height	Population	2,593	22.76	**<0.0001**						
	Herbivore removal	1,593	0.0005	0.98						
	Pop × Herb rem	2,593	0.37	0.69						
Late-season plant height	Population	2,583	46.96	**<0.0001**						
	Herbivore removal	1,583	40.07	**<0.0001**	-6.33	**<0.0001**	-3.10	**<0.0001**	1.18	0.24
	Pop × Herb rem	2,583	14.32	**<0.0001**						
Flowering status	Population	2,586	93.64	**<0.0001**						
	Herbivore removal	1,585	64.85	**<0.0001**	-7.58	**<0.0001**	-4.50	**<0.0001**	0.38	0.71
	Pop × Herb rem	2,584	29.13	**<0.0001**						
# flowers per reproductive plant	Population	2,340	3.45	**0.03**						
	Herbivore removal	1,340	9.93	**0.002**	-3.15	**0.002**	-1.14	0.26	-0.60	0.55
	Pop × Herb rem	2,340	3.14	**0.04**						
# seeds per flower	Population	2,339	3.95	**0.02**						
	Herbivore removal	1,339	8.90	**0.003**	-2.98	**0.003**	-2.12	**0.04**	0.66	0.51
	Pop × Herb rem	2,339	5.31	**0.005**						
# fruits per plant	Population	2,583	56.88	**<0.0001**						
	Herbivore removal	1,583	141.16	**<0.0001**	-11.88	**<0.0001**	-4.85	**<0.0001**	-0.64	0.52
	Pop × Herb rem	2,583	32.65	**<0.0001**						
# seeds per plant	Population	2,583	69.03	**<0.0001**						
	Herbivore removal	1,583	128.25	**<0.0001**	-11.32	**<0.0001**	-5.52	**<0.0001**	0.07	0.95
	Pop × Herb rem	2,583	32.85	**<0.0001**		** **		** **		

**Table 2:** Effects of population and herbivore removal on damage from herbivores and fitness components in three *Lythrum salicaria* populations in the year following the experimental treatment (2014). Flowering status (flowering vs. not flowering) was analysed with a generalized linear model (*χ2* given); the other response variables were analysed with ANOVA (*F*-ratios given). The effect of herbivore removal was analysed separately by population with contrasts when a significant population × treatment interaction was detected. Significant effects are in bold.

					Forsmark		Skagsudden	
Response variable	Source of variation	df	*F / χ2*	*P*	*t*	*P*	*t*	*P*
Proportion of leaf area removed	Population	1,331	451.64	**<0.0001**				
	Herbivore removal	1,331	0.0003	0.99				
	Pop × Herb rem	1,331	0.14	0.71				
Early-season plant height	Population	1,356	46.64	**<0.0001**				
	Herbivore removal	1,356	1.97	0.16				
	Pop × Herb rem	1,356	1.05	0.31				
Late-season plant height	Population	1,333	50.95	**<0.0001**				
	Herbivore removal	1,333	2.01	0.16	-1.42	0.16	1.56	0.12
	Pop × Herb rem	1,333	4.43	**0.04**				
Flowering status	Population	1,335	106.17	**<0.0001**				
	Herbivore removal	1,334	0.11	0.74				
	Pop × Herb rem	1,333	1.25	0.26				
# fruits per plant	Population	1,333	104.20	**<0.0001**				
	Herbivore removal	1,333	1.67	0.20	-1.29	0.20	2.35	**0.02**
	Pop × Herb rem	1,333	6.65	**0.01**				

The effect of herbivore removal on late-season plant height, the number of fruits per plant and female reproductive success and its three components (the proportion of plants flowering, number of flowers per reproductive plant, and number of seeds per flower) in the year of the treatment varied among populations (significant population × treatment interactions, Table 1). Plant height was positively affected by herbivore removal in the southern (1.5-fold increase) and in the central (1.2-fold increase), but not in the northern population (Table 1, Fig 2C). Likewise, female reproductive success increased after herbivore removal in the southern (23-fold increase) and in the central (4-fold increase), but not in the northern population (Table 1, Fig 2H), and two of its components, the proportion of plants flowering and the number of seeds per flower, showed the same pattern (southern population, proportion flowering: 4-fold increase, seeds per flower: 1.6-fold increase, central population, proportion flowering: 2-fold increase, seeds per flower: 1.2-fold increase, northern population: no differences, Fig 2D and 2F). A similar pattern was documented for the number of fruits per plant (southern population: 37-fold increase, central population: 4-fold increase, northern population: no significant effect; Fig 2G). The number of flowers per reproductive plant was positively affected by herbivore removal only in the southern population (5-fold increase, Fig 2E).

Herbivore-removal changed the relative ranking of the populations in terms of plant size and female reproductive success. In the control, plants were 1.4 times taller in the northern population compared to the southern population, whereas after herbivore removal, this difference was reversed; southern plants were 1.1 times taller than northern plants (Fig 2C). Similarly, fruit output per plant in the northern population was 8.1 times higher than that in the southern population in the control, whereas after herbivore removal it was 3.4 times higher in the southern compared to the northern population (Fig 2G), and seed output per plant was 8.6 times higher in the northern population than that in the southern population in the control, whereas when herbivores were removed it was 2.5 times higher in the southern compared to the northern population (Fig 2H).

No significant effect of the herbivore-removal was detected on flowering status in the year following the experimental treatment, but significant population × herbivore removal interactions were detected for late-season plant height and number of fruits per plant (Table 2). In the southern population, control plants tended to be shorter and produce fewer fruits compared to plants from which herbivores had been removed in the previous year, while the opposite was true in the northern population (Fig 3C and 3E). However, contrasts indicated that only the difference in fruit production in the northern population was statistically significant (Table 2, Fig 3D).

Summer survival (from spring to fruit maturation) was very high in all populations (control: range 96–99%, herbivore removal: range 99–100%), and all plants whose markings were found in the spring of 2014 had survived the winter. Cumulative survival until the end of the experiment ranged from 92 to 97% in the control, and from 95 to 100% in the herbivore-removal treatment.

## Discussion

This study has documented considerable variation in damage from insect herbivores and its effects on plant performance among three natural populations of the perennial herb *Lythrum salicaria* located along a latitudinal gradient in Sweden. Both the intensity and effects of herbivory were strongest in the southernmost population, and herbivory was a key determinant of among-population variation in plant stature and fecundity.

The intensity of herbivory was highest in the southern population, Forsmark, and lowest in the northernmost population, Skagsudden. Lower intensity of herbivory towards the north is consistent with a previous study where intensity of herbivory in 12 natural populations of *L*. *salicaria* in Sweden tended to decrease with increasing latitude [14], and also with the decreasing intensity of plant-herbivore interactions away from the equator documented in several other systems [3–7]. The decrease in herbivory is associated with a reduction in the length of the growing season (from about 185 d to 145 d), and with the number of insect herbivores feeding on *L*. *salicaria* [29]. Differences herbivory were apparently not a simple function of variation in plant growth among the three study populations. In the herbivore-removal treatment, plants grew tallest in the southernmost population, suggesting that plant productivity was highest at this site, but the shortest plants were observed in the central rather than the northern population (Fig 2C).

Herbivory negatively affected several components of plant fitness. The effects on plant size, female reproductive success and its three components (probability of flowering, number of flowers per reproductive plant and number of seeds per flower) varied among populations and were strongest in the southern population and intermediate in the central population, while no effects were observed in the northern population. Among-population variation in effects of herbivore removal on plant fitness thus mirrored differences in intensity of herbivory in control plants, and suggests that the strength of herbivore-mediated selection on traits influencing resistance and tolerance to herbivory is positively related to the intensity of herbivory in *L*. *salicaria*. Consistent with this expectation, a previous common-garden experiment documented a positive relationship between resistance against insect herbivory and intensity of herbivory in the source populations [14]. In contrast, in the same study, tolerance to damage increased from south to north indicating that traits influencing tolerance to damage, such as vegetative phenology, vary in response to other environmental factors [14]. Positive correlations between intensity of herbivory and plant resistance to herbivory have been documented in other species [30–32], and a few studies have experimentally demonstrated that selection exerted by herbivores can drive the evolution of among-population variation in resistance [33–35]. The relationship between intensity of herbivory and plant tolerance has rarely been examined, but in a common-garden study on *Iva frutescens* that included genotypes originating from a latitudinal gradient, tolerance to herbivory did not depend on geographic origin or intensity of herbivory in the source populations [15]. Available evidence thus suggests that plant resistance may more commonly be related to intensity of herbivory than is tolerance to damage.

Herbivory strongly affected the relative fecundity of the three populations. In the control, seed output per plant in the northern population was 8.6 times higher in the northern than in the southern population, whereas after herbivore removal it was 2.5 times higher in the southern population compared to the northern. The results suggest that herbivory is a key factor influencing the demographic structure (proportion of flowering plants) and variation in fecundity among *L*. *salicaria* populations in the native range. Strong reductions in fecundity due to herbivory by *Galerucella* spp. have previously been observed in North America, where *L*. *salicaria* is invasive and the beetles have been introduced as biological control agents [36–38]. Additional demographic studies are required to determine the extent to which these strong effects on fecundity translate into reduced population growth rates.

No significant positive effect of herbivore removal were detected on measures of plant performance in the year following the experimental treatment even in the most damaged population Forsmark, in which on average 11% of the leaf area was removed. This suggests that responses recorded in the first year captured the effects of herbivory on plant fitness in the study populations. In some other perennial herbs, negative effects of herbivory in the year following damage have been documented, but the effects have been found to vary depending on timing of damage and the kind of tissue damaged. In *Trillium grandiflorum*, plants consumed early in the season were more likely to regress to non-reproductive stages and become smaller the next season compared to plants consumed late [20]. In *Primula veris*, defoliation early in the season reduced plant size in the following year, whereas damage during fruit development reduced probability of flowering the following year [39]. In *Arabidopsis lyrata*, the effects of experimental damage on performance in the following season varied depending on whether the damage was inflicted on leaves, flowers, or the two combined [21]. However, in these studies, damage levels were in general higher than in the current study (up to 50% defoliation [21,39], or entire plants consumed by deer [20]), and we cannot rule out that a negative effect on performance in the following year could be detected in *L*. *salicaria* populations at higher levels of damage. For a full understanding of how damage to leaves versus reproductive structures and timing of damage affects future performance in *L*. *salicaria*, experimental manipulation of damage levels is needed.

Taken together, the results suggest that damage from native insect herbivores may strongly affect the demographic structure of *L*. *salicaria* populations and thereby shape geographic patterns of seed production. The effects of herbivory on plant fitness increased with increasing intensity of herbivory, and because the number of insect species feeding on *L*. *salicaria* and the intensity of damage they cause tend to decrease towards the northern range margin [14], so should the influence of herbivory on reproductive output and on selection regimes in natural populations of this species.
